# Reduced Clavicle Length Indicates the Severity of Scapular Misalignment in Obstetric Brachial Plexus Lesions

**DOI:** 10.3390/jpm14080846

**Published:** 2024-08-09

**Authors:** Rudolf Rosenauer, Antal Nógrádi, Stefan Quadlbauer, Markus Schmidhammer, Robert Schmidhammer, Savas Tsolakidis

**Affiliations:** 1 Trauma Hospital Lorenz Böhler of the Austrian Workers’ Compensation Board (AUVA), Donaueschingenstraße 13, 1200 Vienna, Austria; rudolf.rosenauer@auva.at (R.R.); stefan.quadlbauer@auva.at (S.Q.); 2 Austrian Cluster of Tissue Regeneration and Ludwig Boltzmann Institute for Experimental and Clinical Traumatology at the Research Centre for Traumatology of the Austrian Workers’ Compensation Board (AUVA), Donaueschingenstraße 13, 1200 Vienna, Austria; m.schmidhammer@gmx.net (M.S.); schmidhammer@millesicenter.com (R.S.); 3 Department of Anatomy, Histology and Embryology, University of Szeged, Kossuth L. sgt 40, 6724 Szeged, Hungary; nogradi.antal@med.u-szeged.hu

**Keywords:** scapular dyskinesia, brachial plexus palsy, clavicle length

## Abstract

(1) Background: Although most brachial plexus birth palsies show some spontaneous recovery, secondary operations are likely to follow. Accordingly, due to the loss of muscle innervation, the growth of the affected limb and the shoulder girdle is reduced. This is associated with pathological scapula positioning and rotation. The objective of this work was to clarify the relationship between length differences of the two clavicles and different types of scapular dyskinesia. (2) Methods: Twenty-five patients suffering from brachial plexus birth palsy were included in this retrospective study. There were eighteen female and seven male patients with a mean age of 10 years (2 to 23 years). CT scans of the thoracic cage, including both shoulder joints and both clavicles, were obtained preoperatively between 2010 and 2012. Radiographic measurements were taken of the axial plane and 3D reconstructions were produced. Functional evaluations of possible movement and scapular dyskinesia were performed. (3) Results: We found an increasing difference in the length of the clavicle (both in absolute and relative terms) in the children with more pronounced scapular dyskinesia. Additionally, with increasing clavicle length differences, the scapula was positioned in a deteriorated angle compared to the healthy side. Significant positive correlations were identified for the age and absolute difference of the clavicle length and the length and width of the scapula on the affected side. (4) Conclusion: Scapular dyskinesia, which is a common finding in brachial plexus birth palsy, is strongly related to reduced clavicle growth. Reduced clavicle length (which is a relatively easily examinable parameter) compared to the healthy side can be used to estimate the extent of scapular malpositioning on the thoracic cage. The extent and severity of scapular dyskinesia increases with augmented differences in the length of the clavicle.

## 1. Introduction

Obstetric brachial plexus lesion (OBPL) is an injury affecting one or more ventral roots or the trunks/divisions of the neonatal brachial plexus [1]. It occurs in about 0.1 to 5.1 cases per 1000 births [2,3]. Although most infants (approximately 90% of the newborns with OBPL) show spontaneous recovery from the primary lesion, a number of OBPL cases require a primary surgery to restore nerve supply of the denervated target muscles [4,5,6]. Despite efforts to re-establish the innervation of the upper arm musculature, in several cases the shoulder girdle muscles remain denervated to some extent, leading to skeleto-muscular maldevelopment [7,8].

The best described maldevelopments are the glenohumeral deformities, including glenoid retroversion and posterior subluxation of the humeral head [9,10]. These deformities are usually associated with the hypoplasia of the scapula and the clavicle [11,12,13]. These maldevelopments are reported to manifest on the basis of disturbed ossification of the bones of the shoulder girdle, thus leading to reduced and distorted growth of the bones. Because these skeletal alterations are thought to be caused by muscular imbalance due to the partial loss of motor innervation, secondary surgeries may be needed to correct this imbalance [14,15,16]. In a healthy fetus, the clavicle is the first bone to ossify by way of membranous ossification from two ossification centers [17]. The onset of fusion of the epiphysis occurs in females at the age of 11 and in males at the age of 14. Although most textbooks suggest complete fusion by 21–22 years of age, as these are the last bones to fuse, some authors suggest that complete fusion does not occur until a maximum age of 26 [18]. Consequently, regulation of ossification and the timeframe for possible adjusting interventions to facilitate physiological bone growth seems to be highly individual.

Empirical experience suggests that the scapula changes its position relative to the thoracic cage during a normal period of crawling when it orientates from a lateral position to a more dorsal one. This process is called retraction of the scapula and it should be completed by the age of one-and-a-half years [19,20,21]. Pathological muscle tone, like in preterm infants or in children with neurological disorders affecting the motor function (i.e., spastic cerebral paresis), is associated with an irregular orientation of the scapula on the thoracic cage [19,20,22].

Similar scapular orientation problems develop in children with OBPL due to muscular imbalances within the shoulder girdle musculature. These conditions result in abnormal rotation and a more lateral orientation of the scapula [23]. In general, the scapulo-thoracic joint contributes to a higher ratio during movement of the shoulder joint in healthy children. Following reduced growth and pathological positioning of the scapula on the thoracic cage, the further range of motion of the scapula on the thoracic cage during movement of the limb is necessary. Moreover, by establishing incorrect innervation patterns such as co-contractions, pathological scapula movements can be triggered by the active motion of adjacent joints, e.g., elbow flexion.

There are several measurements in use to determine the extent of skeleto-muscular alterations in OBPL cases. These usually include determining the length of the clavicle, the dimensions and position of the scapula, and glenoid morphology, all based on CT or MRI imaging. It should be noted that, to our knowledge, the relationship of these valuable parameters to each other has not been analyzed yet.

The aim of this study was to investigate the relationship between the clavicle length difference and the dimensions and the rotation of the scapula in OBPL cases based on CT scans.

## 2. Materials and Methods

We evaluated and examined 25 patients with OBPL who were investigated through the use of a CT scan between 2010 and 2012. All scans were performed in supine position with the arms lying relaxed beside the body and with both palmar surfaces facing the thighs and positioned in a paramedian sagittal plane. In total, 18 female and 7 male patients with a mean age of 10 years (2 to 23 years) were included. Two out of the twenty-five patients had neurolysis before the age of one year. Fourteen of them received a muscle-tendon transfer at various ages (between 2 and 20 years of age). There was a dominance of the right side being affected in 21 cases. The severity of the brachial plexus lesion was graded using the Narakas classification [24]. Accordingly, 6 patients (24%) were assigned to grade I, 13 (52%) to grade II, and 6 (24%) to grade III. No patient met the criteria of grade IV. For detailed patient information, please see Supplemental Table S1.

**Figure 1:**
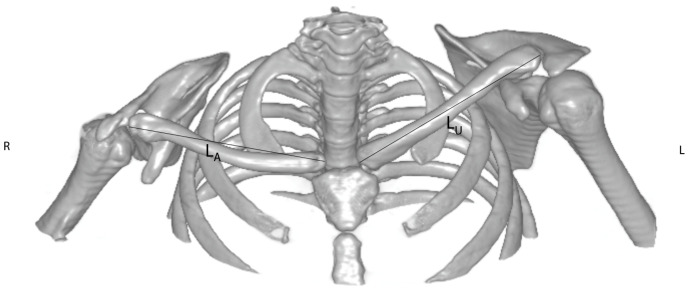
Measurement of the clavicle length based on 3D CT scan reconstruction of the shoulder girdle of an OBPL patient. The antero-superior view of the 3D reconstruction makes it possible to correctly determine the exact length of both clavicles between the medial and lateral ends. Note the considerable shortening of the right clavicle (L_A_ = length on the affected side) compared to the unaffected side (L_U_). For the ratios calculated in the study, the formula length difference = L_U_ − L_A_ was used.

**Figure 2:**
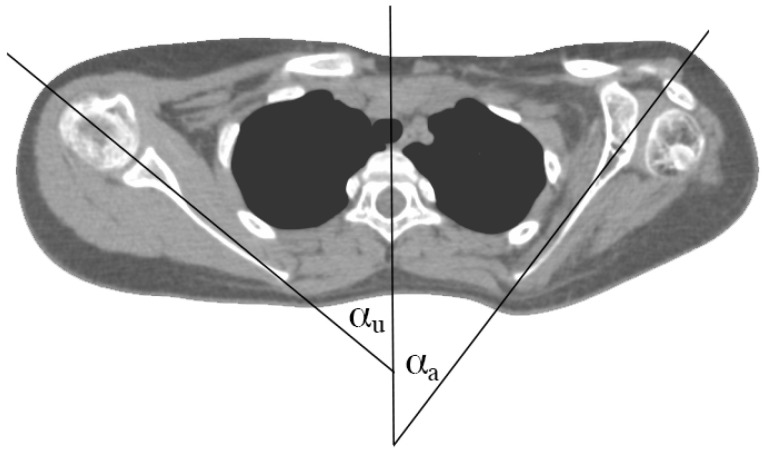
Determination of the lateral orientation angle on a 2D CT slice taken from an OBPL patient. The lateral orientation angle was measured by drawing the midline running through the spinous process and the middle of the vertebra and a line through the scapular axis. The angle given by these two lines is labelled as α_a_ and α_u_ for affected and unaffected sides, respectively. For the ratios calculated in the study, the formula angle difference = α_u_ − α_a_ was used.

**Figure 3:**
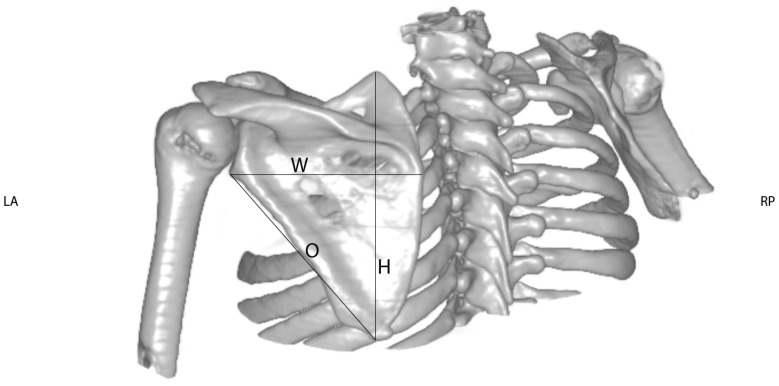
Measurement of the dimensions of the scapula based on 3D CT scan reconstruction of the shoulder girdle of an OBPL patient. A dorso-lateral view, perpendicular to the dorsal surface of the scapula, was chosen to assess the maximum extent of its width and height. Additionally, the oblique length between the inferior angle and the infraglenoid tubercle was measured. All three measurements were obtained for the affected and the unaffected side. Differences were calculated using the formula difference = measurementunaffected − measurementaffected.

Through the use of the 3D volume reconstructions with Osirix MD^®^ software (Version 9.0.1, Pixmeo^®^, Bernex, Switzerland), the length of the clavicle was measured as the maximum extent of the clavicle from the medial to the lateral end taken from an antero-superior aspect (see Figure 1). This view was chosen to correctly determine the exact length of the clavicle, because from this aspect the whole length of the clavicle could be assessed precisely. Thereafter, the length difference between the healthy and the affected sides was calculated. The orientation of the scapula on the thoracic cage was evaluated on CT slices displaying the scapular body. The angle between a sagittal axis drawn through the midline of the vertebral column and the axis of the scapula (represented by a line drawn from the midpoint of the glenoid fossa to the medial end of the scapula) was measured (according to Friedman et al. (see Figure 2). The more acute the angle, the more laterally the scapula was positioned (see Figure 2). The height (H) and width (W) of the scapula were measured using dorsolateral views of 3D volume reconstruction images, where the scapula was looked at perpendicularly to its dorsal surface. As an extra value, the oblique length (O) determined as a distance between the inferior angle and the infraglenoid tubercle was measured (see Figure 3).

**Figure 4:**
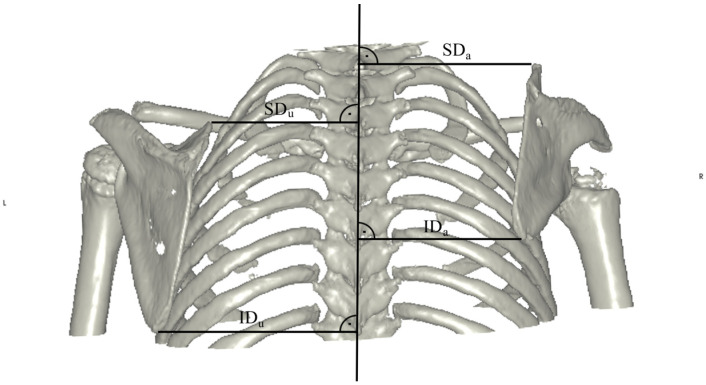
Measurement of the distances of the superior (SD) and inferior (ID) margins of the scapula to a line drawn through the spinous processes of the vertebral column. A strict dorsal view was chosen. Both measurements were obtained for the affected and the unaffected side. Differences were calculated using the formula difference = measurementunaffected − measurementaffected.

**Figure 5:**
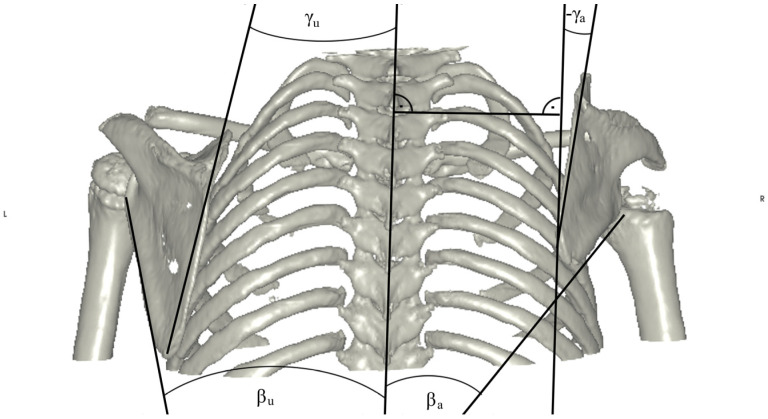
Measurement of the angles of the medial (γ) and lateral (β) borders of the scapula to a line drawn through the spinous processes of the vertebral column. In the case of progressive downward rotation of the scapula, even negative values were measured (see γ on the affected side). Both measurements were obtained for the affected and the unaffected side. Differences were calculated using the formula difference = measurementunaffected − measurementaffected.

To describe the exact position of the scapula relative to the vertebral column, the distances between the superior (SD) and inferior (ID) margins of the scapula and the midline drawn through the spinous processes were measured (see Figure 4). The rotation of the scapula was characterized by the angles between the midline and the medial (α) and lateral (β) margins of the scapula, respectively. In extreme cases, negative values could also be seen (see Figure 5).

The grade of glenoid retroversion and the percentage of posterior humerus head subluxation was measured on axial CT slices according to Waters et al. [25].

Correlations were computed using a two-tailed Spearman test. A univariate ANOVA analysis was performed in order to find differences between patients with or without prior surgery concerning restoration of muscle imbalance. Normal distribution was calculated using the Kolmogorov–Smirnov test. Results were considered to be significant with *p* values < 0.05. All the statistical analyses were performed using IBM^®^ SPSS^®^ Statistics, Version 24.

## 3. Results

First, we investigated whether the patient subgroups (patients with or without surgeries aiming at restoration of muscle imbalance in the shoulder girdle) had differing functional and radiological features at the time of taking their CT scans. No significant functional differences were found between the patient groups with or without surgery through the use of the well-established Mallet score (*p* for Mallet Score = 0.415). Furthermore, no significant radiologic differences were found between these two groups (*p* = 0.324 for the clavicle length difference). These data were further strengthened by finding no significant differences according to the Narakas classification (*p* = 0.872).

Thus, we decided to unite these two subgroups and the 25 patients formed a homogeneous study group. A normal distribution of the clavicle length values on the healthy and the affected sides was calculated, and both groups were normally distributed (*p* > 0.05).

**Figure 6:**
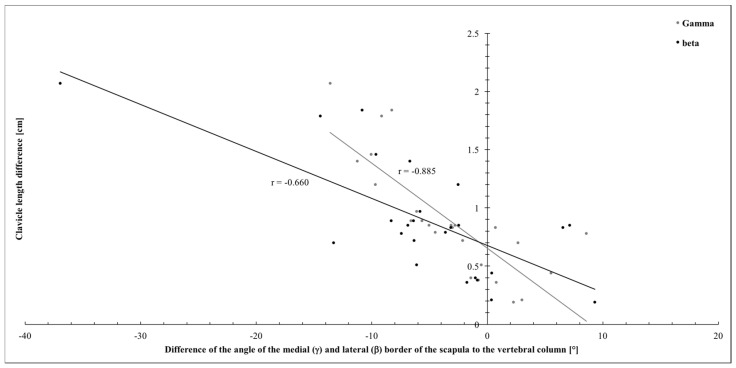
Correlation of the differences of the angles of the medial (γ) and lateral (β) border of the scapula to the vertebral column and the clavicle length differences.

Our previous experience suggested that combination of certain, previously described and widely used parameters may provide valuable information about the extent of skeleto-muscular maldevelopment in OBPL patients. In our cases, the clavicle length difference was correlated with various other parameters. Indeed, significant (strong for α and moderate for β) correlations were found between the clavicle length difference and the angle between the midline and the medial border of the scapula (α, r = −0.885, *p* < 0.001) and the lateral border of the scapula (β, r = −0.660, *p* < 0.001; for details see Figure 6).

**Figure 7:**
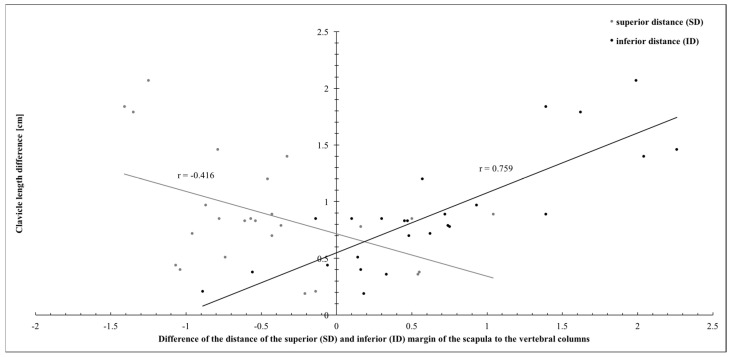
Correlation of the distances of the superior (SD) and inferior (ID) margins of the scapula to the vertebral column and the clavicle length difference.

Again, significant correlations were found between the clavicle length difference and the distance of the superior angle of the scapula to the midline (SD, r = −0.416, weak correlation; *p* = 0.039) and the inferior angle of the scapula to the midline (ID, r = 0.759, moderate correlation; *p* < 0.001; see Figure 7).

**Figure 8:**
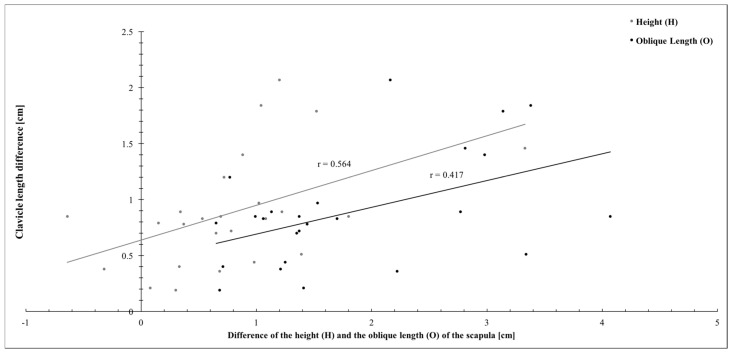
Correlation of the height (H) and the oblique (O) length of the scapula and the clavicle length difference.

When the correlation between the clavicle length difference and the height of the scapula (H) and the oblique length of the scapula (O) was analyzed, only moderate (r = 0.564, *p =* 0.003) and weak (r = 0.417, *p* = 0.038) correlations were seen, respectively (Figure 8). The width of the scapula did not show any significant correlation with the clavicle length difference.

Furthermore, no correlations were found between the clavicle length difference and the grade of glenoid retroversion and posterior humerus head subluxation. All measurements of the different parameters of the affected and healthy shoulder girdles are published in Table 1. All correlations are summarized in Supplemental Table S2.

## 4. Discussion

Our results have provided evidence that reduced clavicle length shows a significant correlation to various extents with abnormal scapular rotation, lateral orientation of the scapula on the thoracic cage, and scapular dimensions in OBPL cases.

Reduced clavicle growth is an important marker in OBPL as it can easily be assessed by clinical examination. It can thus be used as a surrogate marker for other developing deformities that are not obvious upon inspection. We were able to demonstrate that the reduced length of the clavicle in a side comparison can be used to monitor the progression of shoulder girdle growth abnormalities. If there is a relative length gain of the clavicle, this leads to improved positioning of the scapula on the thorax. Of course, there is an increase in the absolute difference in clavicle length due to the growth of the patient, and this difference increases with growth and age. When we compared the ratios of the clavicle length and the width and height of the scapula, we found no significant differences between the three groups, indicating that there was a similarly reduced growth rate in the clavicle and the scapula.

Additionally, no correlations were found between the clavicle length difference and the Narakas grading. The Narakas classification divides brachial plexus lesions based on the affected cervical roots. All grades include a lesion to the roots C5 and C6. Our results suggest that, due to the missing correlation between the skeletomuscular maldevelopment and Narakas grading, the intact roots C5 and C6 seem to be crucial for the development of the shoulder girdle. Accordingly, a lesion to the other cervical roots of the brachial plexus is not likely to be associated with an increased pathologic development of the clavicle and the scapula.

The correct position of the scapula on the thoracic cage is thought to be a prerequisite for normal shoulder function [19,20,26]. Chung et al. [27] has defined the “scapular elevation sign” and the “Putti sign”. In these patients, scapular dyskinesia with a protrusion of the superior border of the scapula through the trapezius muscle can be observed. Furthermore, in the case of a “reverse Putti sign”, scapular dyskinesia occurs in the presence of an external rotation contracture. At early stages of this pathologic development, the scapular malrotation is a dynamically occurring event, for example, during elbow flexion. In the case of ongoing pathologic growth, this dynamic malrotation progresses into a static, pathologic scapula position at the thoracic cage. To the best of our knowledge, no data are available on when this progression occurs. We were able to show that the likelihood of scapula malrotation rises with increasing clavicle length differences and, as a consequence, with the more sagittal orientation of the scapula to the thoracic cage. Due to the lack of available grading systems of scapular dyskinesia, no limits could be determined to discriminate between a scapular dyskinesia and a static, pathologic scapula position at the thoracic cage.

Possible triggers for this dyskinesia could be reduced scapula stabilization or the relatively too strong muscles of the upper arm. Accordingly, the effect of muscle transfers on the behavior of the scapula must be evaluated in further studies.

Scapula retraction occurs throughout normal development [19]. By this process, the angle between the plane of the scapula and a sagittal axis drawn through the midline of the vertebral column increases (see Figure 2). Increased scapular retraction has been linked to abnormal motor development, for example, spastic cerebral palsy. On the other hand, the scapula retraction lags behind following denervation, i.e., due to brachial plexus birth palsy. The decreased angle of the plane of the scapula and the sagittal plane can be seen as a sign of incomplete scapular retraction. Due to this pathologic scapular retraction, the scapula shows a more lateral orientation at the thoracic cage. Reduced clavicle length, which is a more easily examinable parameter, is accompanied by pathologic scapular positioning on the thoracic cage. Whether reduced clavicle growth is the cause or a symptom of this pathologic scapula position is uncertain. The role of scapula stabilizers after brachial plexus birth palsy as a possible therapeutic approach thus needs to be reconsidered in order to clarify the reason for scapula malpositioning.

In children suffering from obstetric brachial plexus palsy, some stages of development are changed or skipped due to muscular weakness. Indeed, according to our unpublished data, a prone position, elevating the thorax, and adequate crawling are not performed properly due to weak elbow extensors. Georgieff and Bernbaum also identified that extensor postural tone and physiologic positioning practices by the baby promote scapular retraction [20]. Our experiences with OBPL children suggest that forced crawling exercises may be a therapeutic approach for promoting scapular retraction.

We included a rather wide range of patients with various ages in our study cohort (range of 2 to 23 years). According to the literature, complete fusion of the epiphysis of the scapula und the clavicle may not occur until the age of 26 [28,29]. According to our results, correlations between the reduced clavicle length and the pathologic scapula position at the thoracic cage were calculated irrespectively of the patients’ age. This supports our thesis of the long-lasting possibilities of potential interventions to increase the growth of the shoulder girdle by improved muscular balance.

We realize that our study has some limitations. Our patient cohort is imperfect due to the restrictions of CT imaging. Naturally, all our measurements and correlations were very much influenced by the different severities of the nerve lesions, regeneration, and interventions prior to our investigations. Most of the elder patients had undergone reconstructive surgeries prior to our study. Of course, data from patients without prior surgery would be preferable. Nevertheless, in order to include a wide range of patient ages, those who had undergone prior surgery had to be part of the study. Furthermore, the measurements of clavicle length can be a critical factor because only the maximum extent was measured. Nonetheless, we accepted this systematic error and decided to work with the percentage differences in clavicle length when comparing the clavicle length and the scapula positioning.

## 5. Conclusions

In this study, we analyzed the relationship between reduced clavicle growth and scapular malpositioning on the thoracic cage and scapular malrotation. A smaller scapula and a shorter clavicle length increase the risk of scapula malrotation and malpositioning on the cage. We were able to demonstrate a clear correlation between the reduced growth of the clavicle and the malpositioning of the scapula. Due to the fact that clavicle length can be measured by palpation of the medial and lateral boarder line, conclusions on the scapula position can be reached, thereby avoiding radiation exposure. By measuring the clavicle length in a side comparison, the effect of surgical and conservative therapies on the morphology of the shoulder girdle can be monitored. If a balance in the length difference can be documented, there is simultaneously an improvement in the positioning of the scapula on the thorax.

## Figures and Tables

**Table 1:** Measurements of the affected and healthy shoulder girdle.

#	Clavicle Length [cm]		Sagittal Orientation of the Scapula [°]		Dimensions of the Scapula [cm]						Distance of the Superior and Inferior Margins to the Vertebral Column [cm]				Angles of the Medial and Lateral Borders of the Scapula to the Vertebral Column [°]			
					Width		Height		Oblique		Superior Margin		Inferior Margin		Medial Border		Lateral Border	
	Healthy	Affected	Healthy	Affected	Healthy	Affected	Healthy	Affected	Healthy	Affected	Healthy	Affected	Healthy	Affected	Healthy	Affected	Healthy	Affected
1	14.29	13.44	40.55	25.93	11.58	10.14	14.67	12.87	12.06	7.99	6.21	6.99	10.47	10.61	13.80	16.94	19.21	21.69
2	8.14	7.36	40.45	32.19	6.98	6.13	7.89	7.52	6.56	5.12	4.54	4.38	7.32	6.57	16.67	8.10	24.49	31.92
3	10.31	9.95	41.33	22.05	9.55	9.09	10.68	10.00	9.48	7.26	7.25	6.71	9.99	9.66	15.49	14.73	12.80	14.58
4	9.84	9.01	40.40	32.97	9.01	8.39	9.72	9.19	7.49	6.43	5.20	5.74	7.99	7.54	25.74	25.05	24.47	17.94
5	6.49	6.28	40.34	23.85	5.69	5.43	6.37	6.29	5.20	3.79	5.22	5.36	6.00	6.89	27.78	24.78	20.47	20.11
6	8.90	8.05	47.51	41.32	7.27	6.76	8.57	9.21	7.18	5.81	4.24	3.74	8.15	8.05	21.37	26.42	11.01	3.88
7	11.13	10.73	44.43	28.91	10.55	8.75	11.58	11.25	9.96	9.25	6.32	7.36	8.23	8.07	5.45	6.89	40.73	41.78
8	8.46	7.76	21.81	16.83	8.03	7.29	9.59	8.94	7.24	5.89	7.54	7.97	8.96	8.48	11.94	9.30	24.04	37.34
9	12.50	11.53	46.55	52.84	12.18	11.17	13.82	12.80	11.28	9.75	6.70	7.57	9.47	8.54	13.59	19.71	23.34	29.18
10	11.06	10.17	41.54	41.67	11.16	10.81	11.58	11.24	9.95	8.82	6.96	5.92	10.51	9.12	17.82	23.47	8.86	17.19
11	10.02	9.64	41.07	35.00	8.25	7.64	10.08	10.40	8.28	7.07	5.61	5.06	8.86	9.42	15.61	16.37	16.22	17.09
12	14.67	12.88	37.78	44.39	13.39	12.70	14.87	13.35	14.56	11.42	7.79	9.14	11.45	9.83	13.98	23.13	8.78	23.23
13	11.53	11.09	41.48	33.03	10.20	9.25	12.38	11.40	11.09	9.84	6.07	7.14	10.56	10.62	20.22	14.72	16.62	16.23
14	9.21	8.38	47.10	35.64	7.75	7.65	9.14	8.06	7.46	5.76	5.87	6.48	8.06	7.59	14.10	17.11	24.37	27.54
15	11.70	10.50	49.37	37.53	9.80	8.90	11.67	10.95	9.38	8.61	8.49	8.95	8.53	7.96	10.03	19.73	33.37	35.89
16	14.66	13.94	61.71	39.88	11.16	10.73	16.92	16.14	14.69	13.32	8.54	9.50	11.12	10.50	6.34	8.49	23.85	30.20
17	11.01	10.50	53.59	48.44	8.31	8.35	11.38	9.99	10.74	7.40	7.67	8.41	8.07	7.93	10.19	10.71	28.08	34.21
18	11.23	9.83	40.87	52.36	10.50	8.96	12.40	11.52	11.42	8.44	6.59	6.92	10.55	8.51	14.91	26.17	14.19	20.89
19	14.28	12.82	39.54	43.60	12.90	12.29	15.57	12.24	13.74	10.93	8.73	9.52	10.51	8.25	8.92	18.97	29.61	39.24
20	7.66	6.81	41.11	36.20	6.32	5.69	8.82	8.13	6.39	5.40	5.53	6.10	7.08	6.78	17.74	20.55	17.98	24.85
21	12.01	9.94	46.48	32.47	9.54	9.14	12.55	11.35	10.77	8.61	7.26	8.51	9.04	7.05	11.37	24.97	26.82	63.77
22	8.82	8.63	35.28	31.09	8.73	7.81	10.31	10.01	8.24	7.56	5.88	6.09	7.70	7.52	12.91	10.66	29.29	19.99
23	12.54	11.75	38.88	34.78	10.01	9.83	12.84	12.69	11.38	10.73	8.63	9.00	9.44	8.70	0.33	4.83	20.38	24.00
24	12.38	10.54	43.43	35.19	9.48	9.20	12.69	11.65	12.36	8.98	7.16	8.57	9.07	7.68	13.23	21.51	23.62	34.47
25	11.79	10.90	48.73	36.97	10.98	10.20	12.36	11.14	10.56	7.79	5.46	5.89	7.64	6.92	12.15	18.75	39.62	46.01

## Data Availability

The data presented in this study are available on request from the corresponding author due to (specify the reason for the restriction).

## Supplementary Materials

The following supporting information can be downloaded at: https://www.mdpi.com/article/10.3390/jpm14080846/s1, Table S1: Detailed patient information; Table S2: Correlation of clavicle length difference with the alignment of the scapula.

## References

[1] Greenhill D.A., Lukavsky R., Tomlinson-Hansen S., Kozin S.H., Zlotolow D.A. (2017). Relationships between 3 Classification Systems in Brachial Plexus Birth Palsy. J. Pediatr. Orthop..

[2] Lalka A., Gralla J., Sibbel S.E. (2020). Brachial Plexus Birth Injury: Epidemiology and Birth Weight Impact on Risk Factors. J. Pediatr. Orthop..

[3] DeFrancesco C.J., Shah D.K., Rogers B.H., Shah A.S. (2019). The Epidemiology of Brachial Plexus Birth Palsy in the United States: Declining Incidence and Evolving Risk Factors. J. Pediatr. Orthop..

[4] Greenhill D.A., Smith W.R., Ramsey F.V., Kozin S.H., Zlotolow D.A. (2019). Double Versus Single Tendon Transfers to Improve Shoulder Function in Brachial Plexus Birth Palsy. J. Pediatr. Orthop..

[5] Leshikar H.B., Bauer A.S., Lightdale-Miric N., Molitor F., Waters P.M. (2018). Clavicle Fracture Is Not Predictive of the Need for Microsurgery in Brachial Plexus Birth Palsy. J. Pediatr. Orthop..

[6] Bahm J., Schäfer B. (2023). Microsurgical Reconstruction of Obstetric Brachial Plexus Palsy: Ongoing Challenges and Future Directions. J. Hand Surg..

[7] van Gelein Vitringa V.M., van Royen B.J., van der Sluijs J.A. (2013). Scapular Deformity in Obstetric Brachial Plexus Palsy and the Hueter-Volkmann Law; a Retrospective Study. BMC Musculoskelet. Disord..

[8] Frich L.H., Schmidt P.H., Torfing T. (2017). Glenoid Morphology in Obstetrical Brachial Plexus Lesion: A Three-Dimensional Computed Tomography Study. J. Shoulder Elb. Surg..

[9] Pearl M.L., Edgerton B.W. (1998). Glenoid Deformity Secondary to Brachial Plexus Birth Palsy. J. Bone Jt. Surg..

[10] Abzug J.M., Wyrick-Glover T.O., Case A.L., Zlotolow D.A., Kozin S.H. (2019). Loss of Midline Function in Brachial Plexus Birth Palsy Patients. J. Pediatr. Orthop..

[11] Russo S.A., Kozin S.H., Zlotolow D.A., Nicholson K.F., Richards J.G. (2019). Motion Necessary to Achieve Mallet Internal Rotation Positions in Children With Brachial Plexus Birth Palsy. J. Pediatr. Orthop..

[12] Pollock A.N., Reed M.H. (1989). Shoulder Deformities from Obstetrical Brachial Plexus Paralysis. Skelet. Radiol..

[13] Dixit N.N., McFarland D.C., Saul K.R. (2019). Computational Analysis of Glenohumeral Joint Growth and Morphology Following a Brachial Plexus Birth Injury. J. Biomech..

[14] Gladstein A.Z., Sachleben B., Ho E.S., Anthony A., Clarke H.M., Hopyan S. (2017). Forearm Pronation Osteotomy for Supination Contracture Secondary to Obstetrical Brachial Plexus Palsy: A Retrospective Cohort Study. J. Pediatr. Orthop..

[15] Lahiji F.A., Tahririan M.A., Karami M., Madadi F., Emami M., Maleki A. (2017). Transfer of Pectoralis Major to Subscapularis in the Management of Brachial Plexus Birth Palsy Sequels. J. Pediatr. Orthop..

[16] DeDeugd C.M., Shin A.Y., Shaughnessy W.J. (2019). Derotational Pronation-Producing Osteotomy of the Radius and Biceps Tendon Rerouting for Supination Contractures in Neonatal Brachial Plexus Palsy Patients: A Review of 20 Cases. J. Pediatr. Orthop..

[17] Pillen S., van Alfen N. (2011). Skeletal Muscle Ultrasound. Neurol. Res..

[18] Jit I., Kulkarni M. (1976). Times of Appearance and Fusion of Epiphysis at the Medial End of the Clavicle. Indian J. Med. Res..

[19] Georgieff M.K., Bernbaum J.C. (1986). Abnormal Shoulder Girdle Muscle Tone in Premature Infants during Their First 18 Months of Life. Pediatrics.

[20] Monfort K., Case-Smith J. (1997). The Effects of a Neonatal Positioner on Scapular Rotation. Am. J. Occup. Ther..

[21] Alberti E.J., Pichorim S.F., Brawerman A. (2019). An Obstetric Brachial Plexus Lesion Rehabilitation Platform. Res. Biomed. Eng..

[22] Sibbel S.E., Bauer A.S., James M.A. (2014). Late Reconstruction of Brachial Plexus Birth Palsy. J. Pediatr. Orthop..

[23] Stein J., Laor T., Carr P., Zbojniewicz A., Cornwall R. (2017). The Effect of Scapular Position on Magnetic Resonance Imaging Measurements of Glenohumeral Dysplasia Caused by Neonatal Brachial Plexus Palsy. J. Hand Surg..

[24] Narakas A.O. (1987). Obstetrical brachial plexus injuries. The Paralysed Hand.

[25] Waters P.M., Smith G.R., Jaramillo D. (1998). Glenohumeral Deformity Secondary to Brachial Plexus Birth Palsy. J. Bone Jt. Surg..

[26] Bahm J. (2016). Upper Limb Multifactorial Movement Analysis in Brachial Plexus Birth Injury. J. Brachial Plex. Peripher. Nerve Inj..

[27] Chung K., Yang L., McGillicuddy J. (2011). Practical Management of Pediatric and Adult Brachial Plexus Palsies E-Book.

[28] Ogden J.A., Phillips S.B. (1983). Radiology of Postnatal Skeletal Development. VII. The Scapula. Skelet. Radiol..

[29] Ogden J.A., Conlogue G.J., Bronson M.L. (1979). Radiology of Postnatal Skeletal Development. III. The Clavicle. Skelet. Radiol..

