# Supplementary material for: Reduced Clavicle Length Indicates the Severity of Scapular Misalignment in Obstetric Brachial Plexus Lesions
Source: J Pers Med. 2024 Aug 9;14(8):846. doi: 10.3390/jpm14080846 (PMC11355126; doi:10.3390/jpm14080846)
Supplement: Supplementary file 1 [file jpm-14-00846-s001.zip › table S2.pdf]

Table S2: Correlation of clavicle length difference to the alignment of the scapula.

**Correlation of clavicle length difference to**	**correlation coefficient, r**	**p**
The angle between the midline of the vertebral column and the		
Medial border of the scapula (α)	- 0.885	< 0.001
Lateral border of the scapula (β)	- 0.660	< 0.001
Distance between the midline of the vertebral column and the		
Superior angle of the scapula (UD)	- 0.416	0.039
Inferior angle of the scapula (ID)	0.759	< 0.001
Height of the scapula (H)	0.564	0.003
Oblique distance of the scapula (O)	0.417	0.038
