# Supplementary material for: Reduced Clavicle Length Indicates the Severity of Scapular Misalignment in Obstetric Brachial Plexus Lesions
Source: J Pers Med. 2024 Aug 9;14(8):846. doi: 10.3390/jpm14080846 (PMC11355126; doi:10.3390/jpm14080846)
Supplement: Supplementary file 1 [file jpm-14-00846-s001.zip › table S1.pdf]

Table S1: Detailed patient information.

#	Age [y]	Sex	Side	Narakas	Primary Plexus Surgery	Secondary muscle-tendon transfer (age [y])
1	15	f	r	2		2 (12)
2	4	f	l	2		n
3	8	m	l	1		1 (6)
4	7	f	r	1	neurolysis	n
5	2	f	r	3		n
6	4	f	r	3	neurolysis	n
7	8	m	r	2		2 (4)
8	7	m	r	3		3 (2), 2 (3)
9	13	f	r	2		n
10	10	f	r	1		2 (6)
11	6	f	r	2		n
12	23	m	r	2		3 (13)
13	15	f	r	3		1 (2)
14	7	f	l	2		2 (2)
15	10	m	r	2		2 (2)
16	20	m	r	2		2 (6)
17	12	f	r	2		n
18	14	f	r	3		5 (18)
19	14	m	r	2		n
20	3	f	r	1		n
21	12	f	r	2		2, 3 (3)
22	9	f	r	3		2 (6)
23	16	f	r	1		n
24	21	f	r	2		5 (5)
25	12	f	l	1		n

1 TMA and LD for external rotation (n = 2)
2 LD for external rotation, TMA and PMA to restore deltoid function (n = 9)
3 Release of internal rotation contracture by PMA and SBSC elongation (n = 3)
4 TMA for external rotation (n = 1)
5 Humerus de-rotation osteotomy (n = 2)
n none (n = 11)
